# Pathology–MRI Correlations in Diffuse Low-Grade Epilepsy Associated Tumors

**DOI:** 10.1093/jnen/nlx090

**Published:** 2017-10-12

**Authors:** Aliya Al-Hajri, Salim Al-Mughairi, Alyma Somani, Shu An, Joan Liu, Anna Miserocchi, Andrew W. McEvoy, Tarek Yousry, Chandrashekar Hoskote, Maria Thom

**Affiliations:** * From The Lysholm Department of Neuroradiology in National Hospital for Neurology and Neurosurgery, London, UK (AA-H, SAM, TY, CH); Division of Neuropathology, National Hospital for Neurology and Neurosurgery, London, UK (AS, SA, JL, MT); Department of Clinical and Experimental Epilepsy, UCL Institute of Neurology, London, UK (AS, JL, AME, MT); and Victor Horsley Department of Neurosurgery, National Hospital for Neurology and Neurosurgery, London, UK (AM, AME).

**Keywords:** Dysembryoplastic neuroepithelial tumors (DNT), Long-term epilepsy associated tumors, MRI

## Abstract

It is recognized that IDH mutation negative, low-grade epilepsy associated tumors (LEAT) can show diffuse growth patterns and lack the diagnostic hallmarks of either classical dysembryoplastic neuroepithelial tumors (DNT) or typical ganglioglioma. “Nonspecific or diffuse DNT” and more recently “polymorphous low-grade neuroepithelial tumor of the young” have been terms used for these entities. There are few reports on the MRI recognition of these diffuse glioneuronal tumors (dGNT), which is important in planning the extent of surgical resection. In 27 LEATs T1, T2, FLAIR, and postcontrast T1 MRI were evaluated and the pathology reviewed, including immunostaining for NeuN, CD34, MAP2, and IDH1. Each case was then independently classified by pathology or MRI as simple DNT, complex DNT, or dGNT. There was agreement in 23/27 (85%; Kappa score 0.62; p < 0.01). In 4 cases, there was discrepancy in the diagnosis of simple versus complex DNT but 100% agreement achieved for dGNT. DNT showed significantly more expansion of the cortex, cystic change and ventricle extension than dGNT. dGNT showed significantly more subcortical T2w hyperintensity and focal cortical atrophy which correlated on pathology with CD34 expression, cortical neuronal loss and white matter rarefaction. There was no distinct cortical dysplasia component identified by MRI or pathology in any case. This study highlights that dGNT can be reliably discriminated on MRI from DNT.

## INTRODUCTION

Dysembryoplastic neuroepithelial tumors (DNT) and gangliogliomas are the most frequent types of long-term epilepsy-associated tumors (LEATs) encountered in epilepsy surgical series (1, 2). Common to both are their low-grade behavior (equivalent to WHO grade I), mixed glioneuronal composition and temporal lobe location, with epilepsy often presenting in childhood to young adulthood (age range in large series at diagnosis: 4–34 years in DNT (3) and 4–33 years in ganglioglioma (4)) that is refractory to medical treatment. DNT have been histologically divided into simple and complex forms, both containing the pathognomonic specific glioneuronal element composed of oligodendrocyte-like cells (OLC) but lacking the dysmorphic ganglion cell component of gangliogliomas (5).

It has long been recognized, however, that a subset of LEATs, representing up to 35% of cases in epilepsy tumor series (1), are difficult to classify microscopically, composed primarily of OLC but showing diffuse, infiltrative, cortical growth patterns, and lacking the specific criteria for either DNT or ganglioglioma (6–11). Although their existence is recognized, they remain “controversial” in the recently revised 2016 WHO classification of CNS tumors (5) and the exact terminology for these lesions in the spectrum of LEATs remains undecided (2, 12). “Nonspecific DNT” (10), “diffuse DNT” (6, 7, 11), “diffuse-oligodendroglial tumor” (13), and more recently “polymorphous low-grade neuroepithelial tumor of the young” (PLNTY) (14) are terms that have been used for what is likely to be the same tumor group. Indeed, pathology descriptions corroborate similar features: Widespread cortical or hippocampal involvement beyond the main tumor mass, CD34 expression and acquisition of degenerative and atrophic changes, as rarefaction and cavitation of the white matter (1, 6, 7, 11, 14). Recent molecular genetic studies have also shown high frequency of mutations in *RAS–RAF–MAPK* and *PI3K–AKT–mTOR* pathways linking this group of tumors (2). For this current study, for simplicity, we adopt the generic term “diffuse glioneuronal tumor” (dGNT) to encompass these lesions. We do not propose this term dGNT as a new nomenclature for these tumors but merely as a “descriptor” to distinguish them from conventional DNT types for the purposes of this study.

There are, however, few studies documenting the MRI characteristics of dGNT. One study highlighted their poor-delineation with grey-white matter blurring and subcortical signal changes and “cortical dysplasia”-like features that contrasted to the cystic/nodular appearances of typical DNT (15). Indeed, the widespread cortical infiltrative nature of dGNT may have led to an overinterpretation of co-existing dysplasia with these tumors (focal cortical dysplasia [FCD]-type IIIb (16)), both by MRI and pathology, accounting for the wide variation in its reporting over the years (6, 9, 17–21). Therefore, improved MRI recognition of dGNT is paramount, both for accurate pre-operative diagnosis and future surgical management when planning the extent of resection, in view of their typically more extensive involvement (22).

The aim of this study was to retrospectively evaluate the MRI features in a recently operated series of LEATs from an epilepsy surgical series to correlate this with histology features and pathology diagnosis of tumor type. We aimed to identify MRI criteria that could reliable discriminate dGNT from DNT as well as assess the frequency of any co-exiting cortical dysplasia.

## MATERIALS AND METHODS

Cases were selected retrospectively through the neuropathology database at the National Hospital of Neurology and Neurosurgery of adult patients who had undergone surgical treatment for management of refractory epilepsy due to an underlying low-grade tumor. The study has ethical approval through the UCL Epilepsy brain and tissue bank (REC12SC0669). We included cases with a histological diagnosis of DNT or LEATs with diffuse growth patterns from 2008 to 2014 where there was consent for research and where optimal pre-operative imaging was available for review. This included availability of the following sequences acquired with 1.5 T or 3 T MR scanners: Sagittal (T1, T2, FLAIR), axial (T2, T2*, T1 post contrast, and DWI, 5 mm thickness), coronal (T2 and FLAIR, 2 mm), and T1 MPR (multiplanar reconstruction) sagittal 1 mm reformatted in axial and coronal plane. We did not include other glioma types in patients without refractory epilepsy and we did not include series of typical gangliogliomas or pediatric gliomas. This selection process resulted in a final group of 27 cases available to study (Table 1). In 19/27 cases interval pre-operative MRI images were available to assess any tumor changes or progression.

**TABLE 1.** Cases Included in the Study, Clinical Data and Localization of the Lesions, Type of Surgical Resection and Regions of Tumor Involvement on Pathological Examination

Case for Table	Age at Time of Surgery (Years)	Gender	Main Location of Lesion on MRI	MRI Diagnosis	Tissues Resected as Samples for Histology	Anatomical Regions of Samples with Tumor	Pathology Diagnosis
1	39	F	L Amyg	COMPLEX DNT	AMYG, Le, TL, HB, PHG, PES, CUSA	Amyg, small focus In TL	COMPLEX DNT
2^a^	26	F	R parietal	SIMPLE DNT	Fragments of Le	All tissue samples	COMPLEX DNT
3	22	F	L superior frontal gyrus	COMPLEX DNT	Fragments of Le	All tissue samples	COMPLEX DNT
4	20	F	R Amyg, HB	COMPLEX DNT	TL, Le, HB, PES, CUSA	ITG, Le, PES	COMPLEX DNT
5	35	M	R PHG, FG, ITG	COMPLEX DNT			COMPLEX DNT
6	61	M	L medial temporal lesion	COMPLEX DNT	TL HB, PHG, PES, CUSA	ITG, HB, PES	COMPLEX DNT
7	42	F	R parietal lobe	COMPLEX DNT	Le, CUSA	In samples of Le	COMPLEX DNT
8^a^	28	M	R medial temporal lesion	SIMPLE DNT	TL and Le		COMPLEX DNT
9	43	F	R MTG	dGNT	Le and CUSA	In samples of Le	dGNT
10	17	F	R FG	dGNT	TL, HB, PES, CUSA (2)	TL only	dGNT
11	31	M	L ITG, FG	dGNT	LE, CUSA	In samples of Le	dGNT
12	48	F	L mesial temporal lesion	dGNT	TL, HB, Le, PHG, CUSA	TL, ITG, HB, Le, PHG	dGNT
13	31	F	R occipital lobe	dGNT	Le	In samples of Le	dGNT
14	16	M	L STG	dGNT	Le and CUSA	In samples of Le	dGNT
15	26	F	R PHG	dGNT	TL, HB	ITG, PHG, HB	^b^dGNT
16	31	M	L PHG	dGNT	TL, HB, PES, Le, CUSA (2)	TL, HB, Le	^b^dGNT
17	26	F	L PHG	dGNT	TL, HB, PHG, PES, CUSA	TL (ITG) HB, PHG, CUSA (NOT PES)	^b^dGNT
18	35	M	R STG	dGNT	TL, HB, PHG, PES, Le, CUSA	TL and Le	^b^dGNT
19	18	M	L Amyg	dGNT	TL, HB, PHG, PES, Le, CUSA	PHG, FG, Le, Temporal pole, HB, PES	^c^dGNT
20	24	M	L MTG	dGNT	Le and CUSA	All tissue samples	^c^dGNT
21	40	M	L PHG	dGNT	TL, HB, PES, Le, CUSA (2)	PHG, HIPPO, TL	^c^dGNT
22^a^	41	M	R MTG	COMPLEX DNT	Le, TL, HB, PHG, PES, CUSA	Le and CUSA	SIMPLE DNT
23	52	F	L frontal rectus gyrus	SIMPLE DNT	Le	In all samples of Le	SIMPLE DNT
24	60	F	R PHG	SIMPLE DNT	TL, HB, PHG, PES, AMYG, CUSA	PHG	SIMPLE DNT
25^a^	31	M	L supramarginal gyrus	COMPLEX DNT	Fragments of Le	In all samples of Le	SIMPLE DNT
26	26	F	R MTG	SIMPLE DNT	Fragments of Le	In all samples of Le	SIMPLE DNT
27	27	M	L STG	SIMPLE DNT	Fragments of Le	In all samples of Le	SIMPLE DNT

The confirmed MRI and pathology diagnosis following review are highlighted in 2 columns. dGNT = diffuse glioneuronal tumor, L = left, R = right, Le = samples from main lesion/lesionectomy, TL = anterior temporal lobectomy (including temporal pole, middle temporal gyrus [MTG], inferior temporal gyrus [ITG] and part of fusiform gyrus [FG], and superior temporal gyrus [STG]), HB = hippocampal body, PHG = parahippocampal gyrus, PES = pes hippocampus, Amyg = amygdala, CUSA = cavitron ultrasonic aspiration samples (either from lesion, amygdala or PHG).

a Indicates cases where there was a mismatch between the pathology and the MRI diagnosis.

b dGNT had a focal ganglioglioma component.

c Cases had a diffuse pattern but with some mixed components of conventional DNT.

### Neuropathological Evaluation

The pathology macroscopic images and histology was reviewed for each case by one neuropathologist (M.T.) on all available histological slides including hematoxylin and eosin (H&E), Luxol fast blue/cresyl violet, and available immunohistochemistry stains including NeuN, neurofilament (SMI32, 31), MAP2, synaptophysin, CD34, *IDH1*, Ki67 and molecular pathology studies including 1p/19q analysis, *BRAF* fusion, *BRAF* V600E mutation, and *ATRX* mutation (Supplementary Data Table S1). All cases had been initially diagnosed (in 25/27 cases by more than one other neuropathologist) as DNT, diffuse DNT or mixed DNT + ganglioglioma; there were no changes to diagnosis following review. For each case, the presence or not of 16 specific or defining as well as other histological features (Supplementary Data Table S2) were recorded. Confirmatory diagnosis of simple and complex DNT was based on WHO criteria (5) and for dGNT on previously developed histological criteria for diffuse DNT (6): (i) diffuse cortical infiltration pattern predominates, (ii) relative lack of glioneuronal element, myxoid matrix, or nodular growth pattern, (iii) underlying white matter often shows rarefaction and cystic atrophy, (iv) tumor cytology is predominantly OLC, and (v) CD34 expression is often present in lesion and perilesional cortex.

### MRI Assessment

The MRI were then evaluated in a two-step process blinded to the pathological diagnosis: First, the presence or absence of 25 specific features in each case was recorded. These included location, size, shape, margins, cysts, calcification, hemorrhage, enhancement pattern, bone remodeling, signal intensity, peri-lesional signal change, contact with the ventricles, multi-gyral involvement, cortical expansion, or atrophy (Supplementary Data Table S2). Second, the cases were then classified as one of 3 possible entities based on current literature and the relative presence or absence of the above features, for example, (i) simple DNT: Single well-defined lesion, cystic components, and cortical expansion; (ii) complex DNTs: Multicystic or “bubbly” appearances; (iii) diffuse GNTs: Perilesional white matter signal changes and focal cortical atrophy. Each case was categorized following a consensus discussion and agreement between 3 neuroradiologists on the imaging features (A.A., S.M., C.H.).

### Correlation of MRI and Neuropathology

A comparison between the MRI derived diagnosis and the neuropathological gold standard was performed and the frequency of each of the MR features in each of the 3 tumor entities was established. Statistical analysis using SPSS (IBM, version 21; Spearman’s correlation, Kruskal–Wallis and Mann–Whitney tests for nonparametric data) was carried out to compare the level of agreement between radiological and pathological diagnosis. Significant differences in the presence of each radiological criteria between tumor groups were assessed to evaluate the relative specificity of MRI features in discriminating dGNT from DNT types. In addition, correlation between MRI and pathology features was carried out.

## RESULTS

### Neuropathology and Molecular Genetics

The pathology review diagnosis confirmed simple DNT in 6 cases, complex DNT in 8 cases, and dGNT in 13 cases (Table 1). Molecular pathology in the dGNT confirmed that none had an *IDH1* mutation, and in 6 cases where *ATRX* mutation had been evaluated, this was not present. Ten cases were investigated for 1p/19q chromosome LOH as part of the routine work-up but this was not present in any. Analysis for BRAF V600E mutation, however, was confirmed in 5/11 dGNT compared with 0/5 complex DNT and 0/3 simple DNT; this included 1/3 cases of dGNT with focal aggregates of ganglion cells (see below).

### MRI Agreement and Features

**FIGURE 1.**
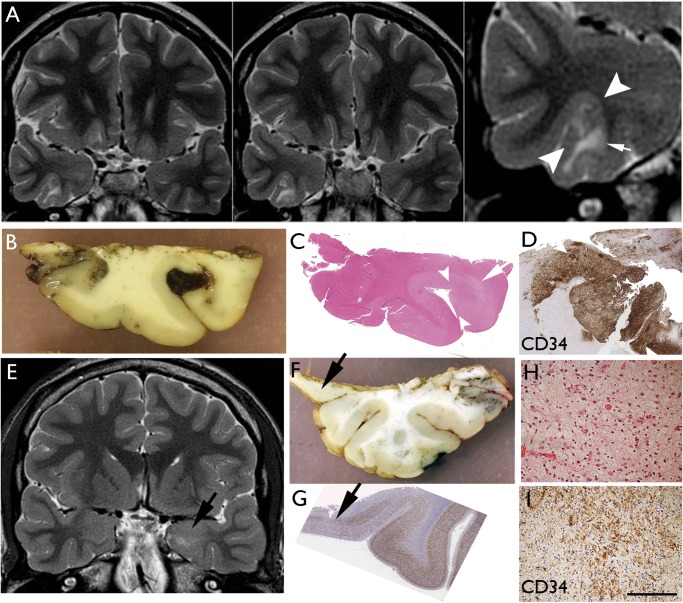
Diffuse GNT. Case 10 **(A–D)** and Case 19 **(E–I)**. **(A)** Coronal T2 weighted images showing cortical based lesion of high signal intensity with subcortical white matter involvement involving the right fusiform gyrus. Note the cortical part of the lesion that is creating a bridge into the adjacent sulcus (between arrowheads) shown in higher magnification. There is signal change in the white matter (arrow). **(B)** Macroscopic image of fixed resection specimen with the slice corresponding to the lesion seen on MRI; there is focal peri-operative acute hemorrhage into the expanded gyrus. **(C)** H&E-stained section indicates the infiltrated gyrus (between arrows) and underlying rarefaction in the white matter correlating with loss of myelin and axons (arrow). **(D)** CD34 of the same gyrus at high magnifications shows the diffuse cellular infiltration of the cortex bridging over to the next gyrus. Case 19: **(E)** dGNT (which also had areas of mixed complex DNT, not shown here) with lesion in MRI in the amygdala (arrow). **(F)** In the temporal lobe resection, diffuse infiltration was confirmed in the inferior-mesial part of the cortex (arrow). **(G)** Depletion of neurons seen in this region of diffuse tumors infiltration on NeuN stain (arrow). **(H)** Diffuse tumor infiltration in the grey matter from the tissue of the lesion in the amygdala. **(I)** This same region shows CD34 positivity of tumor cells. Bars: **D** = 500 μm; **H, I **=** **∼300 μm.

Twenty-two tumors were located in the temporal lobe, 2 in the frontal, 2 in the parietal and one in the occipital lobe (Table 1). The MRI diagnosis was simple DNT in 6 cases, complex DNT in 8 cases (Supplementary Data Fig. S1) and dGNT (Fig. 1) in 13 cases. The MRI diagnosis was confirmed neuropathologically in 85% (23/27) of cases (Kappa score 0.62; p < 0.000). There was 100% (13/13) agreement between the MRI and neuropathological diagnosis of dGNT (Table 1). In simple DNT, there was a 67% (4/6) agreement with 2 cases incorrectly diagnosed as complex DNT. In complex DNT, there was a 75% (6/8) agreement with 2 cases wrongly diagnosed as simple DNT (Table 1). In 2 of the cases with lack of agreement, only small resection samples were available for histology diagnosis.

**FIGURE 2.**
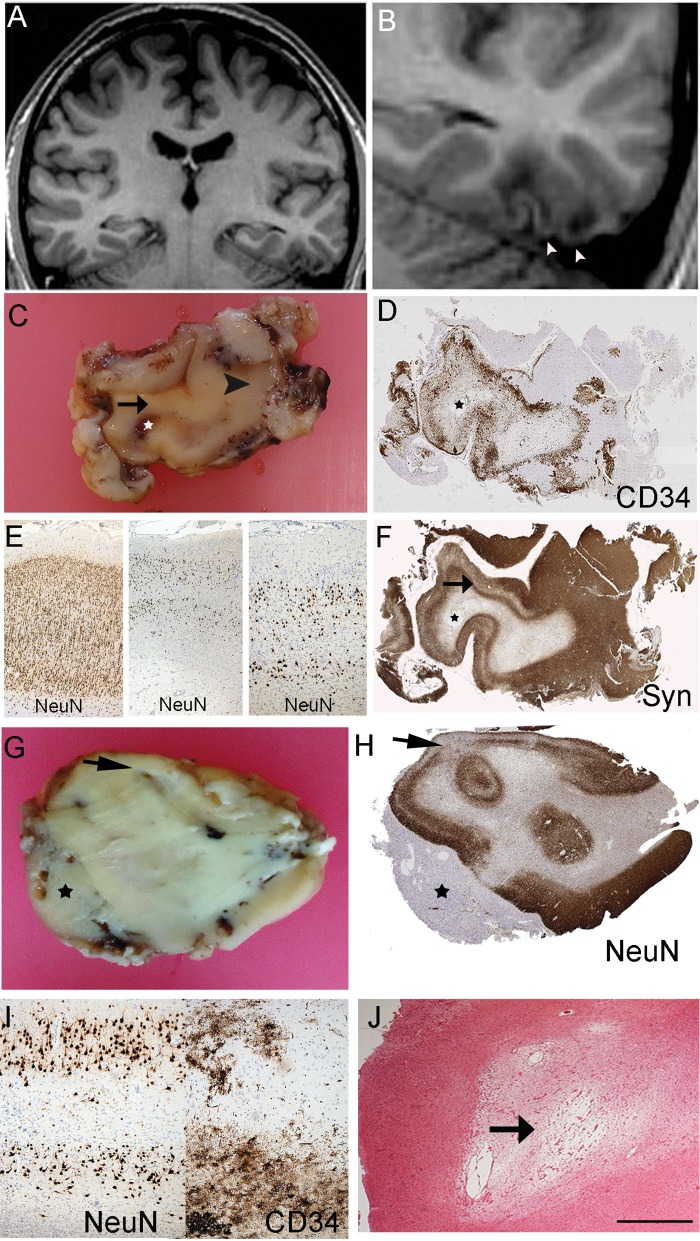
Diffuse DNT with cortical and white matter atrophy. Case 11 **(A–F)** and Case 9 **(G–J)**. **(A)** Coronal T1 weighted MR images showing a lesion involving the left fusiform and inferior temporal gyri. **(B)** The lesion is shown at higher magnification with cortical atrophy with underlying white matter rarefaction in the region of tumor infiltration as well as apparent overlying retraction of cortex and a superficial CSF filled space. **(C)** The fixed resected specimen showing atrophic discolored cortex (arrowed) and underlying cavitated white matter (star in each image); arrowhead indicates region of normal cortical thickness. **(D)** CD34 confirmed extensive cortical tumor infiltration in this region. **(E)** Panel of NeuN showed, on left laminar cortex in preserved region (arrowhead in **D**) compared with middle and right images of tumor infiltration region with laminar cortical atrophy in region of infiltration. **(F)** Synaptophysin also highlights laminar neuronal loss in tumor infiltration region (arrow) imparting an impression of cortical “collapse” rather than expansion. Case 9: **(G)** Macroscopic section of diffuse GNT with evidence of cortical atrophy (arrow) and extension into the leptomeningeal tissues forming a mass (asterisk) and with poor definition between grey and white matter. **(H)** NeuN on a section from **G** confirms the thinning and laminar atrophy of the cortex (arrow) and the lack of a ganglion component in the leptomeninges (asterisk). **(I)** Higher magnification of the region shown by arrow in **(H)** showing laminar neuronal loss and on CD34 in the same region evidence of tumor infiltration. **(J)** H&E stain shows focal cavitation and rarefaction of white matter underlying the infiltrated cortex. Bar in **J **=** **∼400 μm.

Features that more significantly associated with dGNT was evidence of ill-defined subcortical white matter T2-w hyperintensity, present in all cases (100%; p < 0.0001; Fig. 1A) and cortical atrophy found in 92% cases (12/13) with thinning in the region of tumor infiltration and retraction with focal prominence of the overlying sulcus forming a CSF filled space (Fig. 2A, B;Table 2; p < 0.0001). For simple DNTs, single well-defined lesions, cystic components, cortical expansion and high signal rim on FLAIR were all more common than in dGNT (Table 2). Complex DNTs demonstrated more frequent multicystic or “bubbly” appearances (p < 0.001; Supplementary Data Fig. S1A, B), with 75% also demonstrating a wedge-shaped lesion (p < 0.001; Supplementary Data Fig. S1G) or multi-gyral involvement (p < 0.05) compared with other tumors. Half of the complex DNTs also showed mass effect and bone remodeling. The presence of a multicystic or “bubbly” appearance was the only significant radiological difference between simple and complex DNT (p < 0.01).

**TABLE 2.** Frequency of Each Feature in the 3 Subtypes of LEAT: Simple, Complex DNT and Diffuse Glioneuronal Tumor (GNT)

Radiological Features	Simple DNT % (Number of Cases	Complex DNT % (Number of Cases	Diffuse GNT % (Number Of Cases	p Value #	Pathology Feature	Simple DNT % (Number of Cases	Complex DNT % (Number of Cases	Diffuse GNT % (Number of Cases	p Value #
Expansion of the cortex	50% (3/3)	75% (6/8)	8% (1/13)	0.026*	Macroscopic cysts	0% (0/6)	25% (2/8)	8% (1/13)	0.4
Multi-gyral involvement	33% (2/6)	75% (6/8)	38% (5/13)	0.023	Microscopic cysts	33% (2/6)	75% (6/8)	15% (2/13)	0.06
Cystic components	50% (3/6)	88% (7/8)	62% (8/13)	0.03*	Distinct nodules	17% (1/6)	100% (8/8)	38% (5/13)	0.005*
Calcification	0% (0/6)	0% (0/8)	46% (6/13)	0.018	Nodules within tumor	17% (1/6)	0% (0/8)	70% (9/13)	**0.006**
Hemorrhage	0% (0/6)	13% (1/8)	23% (3/13)	0.42	Glioneuronal element	100% (6/6)	100% (8/8)	8% (1/13)	0.000*
Mass effect	33% (2/4)	50% (4/8)	15% (2/13)	0.49	Ventricle extension	17% (1/6)	25% (2/8)	0% (0/13)	0.3
Bone remodeling	0% (0/6)	50% (4/8)	0% (0/13)	**0.005**	Mucoid matrix	67% (4/6)	100% (8/8)	0% (0/13)	**0.000**
Single well-defined lesion without abnormal signal around it	50% (3/6)	25% (2/8)	0% (0/13)	0.001*	Diffuse growth-predominant pattern	0% (0/6)	0% (0/8)	84% (11/13)	0.000*
Multicystic	0% (0/6)	100% (8/8)	36% (4/11)	**0.001**	Dysmorphic neurons absent	100% (6/6)	88% (7/8)	38% (5/13)	**0.002**
“Bubbly” appearance	33 (2/6)	88% (7/8)	8% (1/13)	0.001*	Calcification	0% (0/6)	12% (1/8)	38% (5/13)	0.65
Perilesional/subcortical white matter signal change	0% (0/6)	13% (1/8)	100% (13)	0.000*	Reticulin rich regions	0% (0/6)	0% (0/8)	15% (2/13)	0.3
Wedge shaped lesion	17% (1/6)	75% (6/8)	8% (1/13)	**0.001**	Pigmentation (marked or focal)	17% (1/6)	75% (6/8)	77% (10/13)	0.033
Focal high signal rim on FLAIR	100%	88% (7/8)	62% (8/13)	0.137	White matter changes:				0.017
					Marked rarefaction	0% (0/6)	0% (0/8)	30% (4/13)	
					Subtle myelin loss	0% (0/6)	25% (2/8)	38% (5/13)	
Focal cortical atrophy with prominent adjacent sulcus	0% (0/6)	12.% (1/8)	92% (12/13)	0.000*	Adjacent cortex layer I hypercellular	0% (0/6)	0% (0/8)	54% (7/13)	0.02
Enhancement	0% (0/6)	25% (2/6)	46% (6/13)	0.055	Dyslamination beyond tumor infiltration zone	0% (0/6)	0% (0/8)	0% (0/13)	0.15
Close contact with ventricle	33% (2/6)	75% (6/8)	15% (2/13)	0.026	CD34 tumoral expression	0% (0/6)	25% (2/8)	100% (13/13)	**0.000**

* These features were used as initial defining criteria to classify the cases. There were no statistically significant differences between simple and complex DNT. Significance is shown for each feature in discriminating between DNT and diffuse GNT subtypes (K–Wallis test); significant values shown in bold as p < 0.01. The presence of rosettes, eosinophilic granular bodies, Rosenthal fibers, necrosis, or mitotic activity was noted in 0–15% of cases, similar across all tumor groups and therefore not further analyzed.

In none of the DNT or dGNT in this series was there evidence either on MRI or pathology of a separate or associated distinct area of FCD in addition to the tumor.

### MRI: Pathology Statistical Correlations

Of the MR and pathology features listed in Table 2, 15 significant positive correlations were noted (p < 0.01, Spearman’s correlation). A “bubbly” appearance on MRI correlated with a mucoid matrix pathologically (p < 0.001; Supplementary Data Fig. S1E) and the presence of a glioneuronal element (p < 0.0001). The presence of calcification on MRI correlated with histological evidence of calcium deposits (p < 0.0001). Subcortical white matter T2w hyper intensity correlated with a predominant histological diffuse growth pattern (p < 0.0001), CD34 expression (p < 0.0001; Figs. 1D, I, 2D, H) and pathological evidence of white matter rarefaction/loss of myelin (p = 0.013; Figs. 1C, 2C, J). Cortical atrophy on MRI with prominence of the adjacent sulcus correlated with diffuse histological growth pattern (p < 0.0001) and the pathology showed evidence of cortical neuronal loss, laminar atrophy, with “collapse” of the cortical ribbon rather than expansion (Fig. 2).

The MRI features that were significantly more common in classical DNTs when compared with dGNT were expansion of the cortex (p < 0.05), the presence of bone remodeling (p < 0.01), multi-cystic or “bubbly” appearance (p = 0.001), well-defined lesions lacking abnormal surrounding signal change (p < 0.001) and close contact or “tail-like” extension toward the ventricle (p < 0.05); the latter being a finding in 63% of complex DNT (Supplementary Data Fig. S1G; Table 2).

**FIGURE 3.**
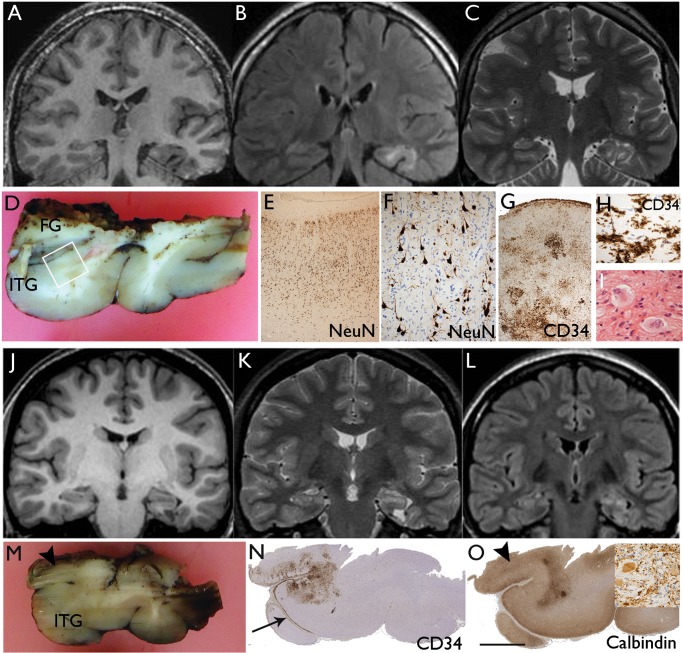
Diffuse GNT with focal gangliogliomatous component. Case 16 **(A–H)** and Case 17 **(J–O)**. **(A)** Coronal T1, **(B)** FLAIR, and **(C)** T2 weighted MR images showing apparent cortical atrophy underlying white matter signal abnormality with multigyral involvement by the tumor. **(D)** The main temporal lobe resection specimen included the middle temporal gyrus the inferior temporal gyrus (ITG) and part of the fusiform gyrus (FG). The ITG (shown in square) was infiltrated by a diffuse tumor: **(E, F)** The same region at higher magnifications shows infiltration of the cortex, reduced neuronal density being “displaced” by NeuN-negative oligodendrocyte-like cells which distort the neuronal morphology. **(G)** Higher magnification of this same region showing clusters of CD34-positive tumor cells in the cortex and cortical layer I; these are shown in **H** and in high magnification in **I.** A sample from the main lesion in the amygdala contained a small focus with atypical gangliod cells. Case 17: **(J)** Coronal T1, **(K)** T2, and **(L)** FLAIR weighted MR images showing left temporal lobe lesion centered on the parahippocampal gyrus with perilesional signal abnormality and cortical atrophy in the adjacent temporal lobe. **(M)** The main temporal lobe resection including the inferior temporal gyrus (ITG) showed microscopic evidence of diffuse infiltration as shown on CD34 stain in **(N)**; note the widespread labeling of CD34-positive cells along subpial border in layer I (arrow). In deeper sections, a focus of atypical ganglion cells was noted in the superior aspect at the inferior-medial resection margin (arrowhead); **(O)** this correlated with calbindin positivity and synaptophysin-positive atypical gangliod cells are shown in this region in the inset. Bars: **F** = 250 μm; **G, H, N (inset)** = ∼60 μm.

**FIGURE 4.**
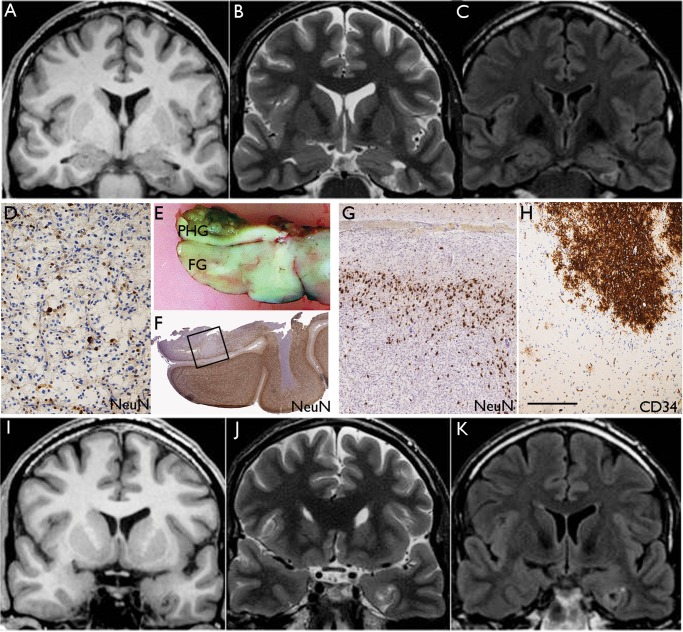
Diffuse GNT with mixed features of DNT. Case 21: A lesion centered in the left parahippocampal gyrus. **(A)** Coronal T1, **(B)** T2, and **(C)** FLAIR MR images showing the lesion involving the left parahippocampal gyrus with a triangular wedge-shaped appearance and a multiple cystic appearance. The wedge-shaped lesion points toward the temporal horn of the left lateral ventricle and the appearance of the tumor in this region resembles the complex subtype of DNT. **(D)** Tissue from the main lesion showed glioneuronal element with floating neurons characteristic of DNT on NeuN stain. **(E)** In the main temporal lobe specimen, which included the fusiform gyrus (FG) and part of the parahippocampal gyrus (PHG), there was diffuse pattern of tumor infiltration which was the dominant overall growth pattern as shown on NeuN stain in **(F)**. The region shown in the square is shown at higher magnification in **(G)** and rotated 180°. **(H)** The infiltrating cells showed patchy CD34 labeling. **(I)** Coronal T1, **(J)** T2, and **(K)** FLAIR corresponding to the level shown in **(E)** confirmed an ill-defined white matter signal abnormality with cortical atrophy; this part of the lesion resembled diffuse GNT. Bars: **D** = 100 μm; **G, H **=** **∼175 μm, based on original magnifications.

In 4 dGNT (cases 15–18), aggregates of atypical ganglion cells were also noted on histology as a minor component, suggestive of focal gangliogliomatous differentiation (Fig. 3) but no specific features distinguished these cases on MRI. In 3 of the dGNT (cases 19–21), although the diffuse pattern predominated, the histology also showed focal regions with a more typical DNT growth pattern or focal glioneuronal element. The MRI in these 3 cases also showed features of dGNT and a minor component more in keeping with DNT, suggesting that some cases may have “hybrid” features (Fig. 4).

### MRI: Progressive Changes

Nineteen of the twenty seven cases had been followed up pre-operatively with a second MRI, the interval period ranging from 1 to 80 months. None of the simple or complex cases showed a change in appearance. However, 3 of the diffuse cases showed change in size and appearance; one showed further atrophy while 2 showed an increase in size.

## DISCUSSION

In this study of a series of operated LEATs, we have shown good agreement between pathology and MRI diagnosis. We have identified MRI features that associate with dGNT to enable its distinction from the typical forms of DNT. In particular, the presence of peri-lesional signal changes and cortical atrophy within the tumor zone were MRI features that were frequent in dGNT and correlated with cortical laminar neuronal loss and white matter rarefaction in the region of diffuse tumor infiltration. Although some MRI features in typical DNT may be indicative of its underlying developmental origin, as the ventricular extension “tail sign,” accompanying FCD was not confirmed by MRI or pathology in this LEAT series.

There have been relatively few published studies directly correlating MRI with pathology in DNT and DNT-like lesions. Campos et al retrospectively evaluated a series of 61 simple and complex DNT (23). They confirmed a high percentage of cases with pseudocysts or smaller cysts with “septations” (akin to the “bubbly appearance” in our descriptions) that correlated with the specific glioneuronal element. In the current study, we also confirmed this finding, with all complex DNT showing multicystic features on MRI and a strong correlation between “bubbly” appearance on MRI and a myxoid extracellular matrix of the glioneuronal element on pathology. In their study, contrast enhancement was present in 21.6% which was comparable to our series (present in 25% of complex DNT; Table 2). They also noted that the distinction of simple from complex DNT was often not feasible by MRI with the only statistically significant discriminating features being the presence of calcification or hemorrhage in the complex form (23). In the current series, the only feature that discriminated between simple and complex tumors was the presence of a multicystic and “bubbly” appearance. Furthermore, the 4 cases with pathology/MRI disagreement in this study were between the simple and complex subtypes. Although this may be partly explained by small tissue sample size in 2 cases, it calls to question that simple and complex may represent a spectrum of the same tumor and questions the rationale to discriminate these in the WHO classification of CNS tumors (5).

We showed 100% agreement between the MRI and histology diagnosis of dGNT, supporting its distinction from conventional DNT within the LEAT tumor group. The only previous MRI/pathology correlative study of diffuse LEAT tumor types noted their poor-delineation on MRI with grey-white matter blurring, subcortical signal changes and frequent location in mesial temporal lobe (15); this MRI appearance was termed “dysplastic-like” when compared with the cystic/nodular appearances of typical DNT. Despite the frequent recognition of “dGNT” types in epilepsy surgical series, these tumors continue to be controversial, masquerading under a range of diagnostic labels over the years from “diffuse/nonspecific DNT” to the more recent “PLNTY” (7, 11, 14, 21). Although lagging behind recent progress in conventional gliomas (2), future advances in the molecular genetic characterization of LEATs (13, 14, 24, 25), including DNA methylation profiling, will undoubtedly advance the much-needed revisions in the nomenclature, including the accommodation and integration of dGNT into the WHO classification (1, 12). In this study, an in-depth molecular analysis was not carried out to further classify these tumors which is a limitation. Recent studies highlight tentative biological linkage of dGNT with DNT through genetic abnormalities in *FGFR1* (13, 24) and some tumors (including cases in the present study) showing hybrid histological features of dGNT and DNT. There is more compelling evidence, however, grouping dGNT with ganglioglioma through the shared and predominant expression patterns of CD34, observation of foci of atypical ganglion cells (including cases in the present study) more frequent *BRAF* V600E gene mutations than DNT and methylation cluster analysis (14, 25, 26). Future molecular studies on large series of LEATs will likely confirm the molecular classification of dGNT as well as the nature of tumors with hybrid features of more than one WHO tumor type (27–32).

In the current study, the most frequent MRI features of dGNT were peri-lesional T2 signal change and cortical atrophy within the tumor zone; these correlated with degenerative features on pathology, including cortical laminar atrophy and white matter rarefaction with axonal and myelin loss. These superimposed “atrophic” features could be an effect of the seizures themselves or attest to the slow growth rate and long-standing nature of the dGNT and is in keeping with the documented accumulation of degenerative changes reported, including TDP43, tau and p62 (6, 33). We failed, however, to confirm co-existing FCD by MRI or histology. FCD associated with LEAT was recently assigned to FCD type IIIb in the ILAE classification (16). The incidence of FCDIIIb, however, varies greatly between series; for example, in the published MRI/pathology correlative studies of DNT, co-existing FCD was reported in 0/61 cases (23) compared with 25/78 (32%) (15). This is likely due to a lack of clear criteria for FCDIIIb (1, 16, 18, 34) and distinction from tumor infiltrative regions (22), and it remains a contentious but important area, including contribution to epileptogenicity (34). However, the striking “wedge” or funnel shape appearance of DNT noted on MRI (23, 35) was present in 75% of our complex DNT and association or contact with the ventricle in 63%, reminiscent of the transmantle/“tail sign” of FCD IIB. A developmental origin of DNT from the peri-ventricular region and germinal matrix has been previously proposed (36).

The advances in the molecular characterization of LEATS, as well as providing diagnostic and prognostic biomarkers, will undoubtedly herald a future of individualized treatment plans including tailored surgery (37–40). The accurate recognition of dGNT on pre-operative MRI and their distinction from other LEATs as well as the more common diffuse low-grade gliomas in adulthood will remain an integral component for appropriate surgical planning. This has particular relevance in dGNT in terms of defining the boundaries of the lesion compared with the epileptogenic zone (9, 15, 41). This study is limited in that it did not carry out full molecular characterization of tumors, correlate MRI lesion with EEG for delineation of epileptogenic zone or compare dGNT with conventional low-grade gliomas or typical gangliogliomas. These would all be important to address in future studies of dGNT to further refine diagnostic criteria and similarities and differences between tumor types. Nevertheless, this study has highlighted the potential of conventional MRI to distinguish dGNT for the future pre-operative identification of this common LEAT subtype. The diagnostic validity of these features will have to be assessed in a prospective study.

## Supplementary Material

Supplementary Data
