# Supplementary figures and images for: Pathology–MRI Correlations in Diffuse Low-Grade Epilepsy Associated Tumors
Source: J Neuropathol Exp Neurol. 2017 Oct 12;76(12):1023–33. doi: 10.1093/jnen/nlx090 (PMC5939705; doi:10.1093/jnen/nlx090)

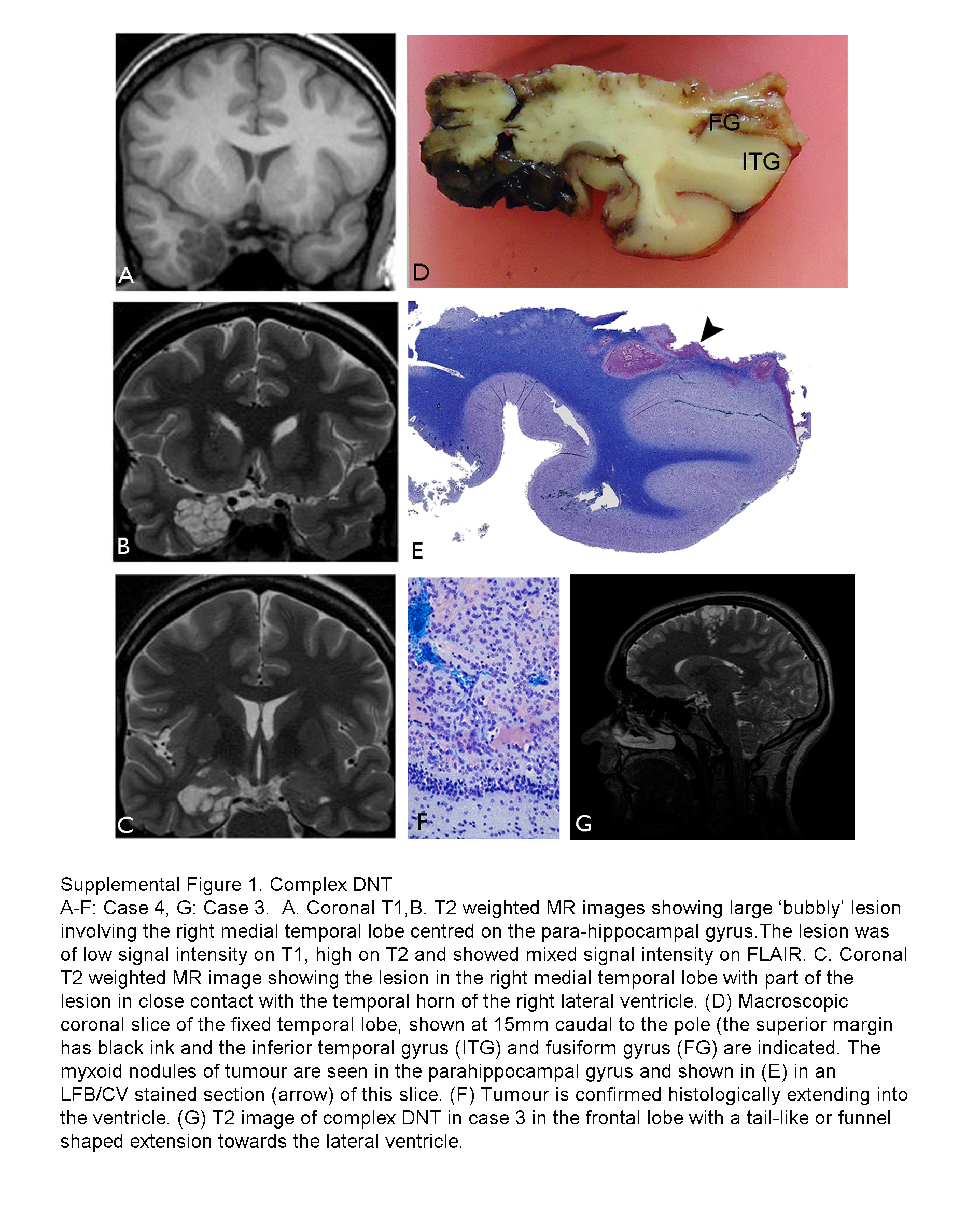

Supplement: Supplementary Data [file nlx090_supp.zip › nlx090_Supp.jpg]
