# Supplementary material for: Pathology–MRI Correlations in Diffuse Low-Grade Epilepsy Associated Tumors
Source: J Neuropathol Exp Neurol. 2017 Oct 12;76(12):1023–33. doi: 10.1093/jnen/nlx090 (PMC5939705; doi:10.1093/jnen/nlx090)
Supplement: Supplementary Data [file nlx090_supp.zip › nlx090_Supptable1.doc]

**Supplemental Table 1: Automated Immunohistochemistry and Molecular Tests**

| **Primary antibody** | **Antibody clone (source)** | **Dilution** | **Antigen Retrieval buffer (mins)** |
| --- | --- | --- | --- |
| NeuN, Neuronal marker | A60 (Millipore) | 1:2000 | ER1 (20) |
| Neurofilament, phosphorylated | SMI31 (Sternberger Monoclonals) | 1:5000 | None |
| Neurofilament, non-phosphorylated | SMI32 (Sternberger Monoclonals) | 1:500 | ER2 (20) |
| MAP2, Microtubule associated protein 2 | HM-2 (Sigma) | 1:5000 | ER1 (20) |
| Synaptophysin | DAK-Synap (DAKO) | 1:100 | ER2 (20) |
| CD34 | QBEnd/10 (Leica Biosystems) | 1:50 | None |
| IDH1 | H09 (Dianova) | 1:64 | ER2 (20) |
| Ki67 | Mib-1 (DAKO) | 1:200 | ER2 (30) |
| ATRX | Polyclonal (Sigma) | 1:500 | ER2 (20) |
| BRAF V600E | VE1 (Spring Bioscience) | 1:50 | ER2 (30) |

These immunomarkers were used to discriminate DNT from dGNT, to identify regions of cortical infiltration and the molecular panel to distinguish dGNT from conventional low-grade gliomas, including IDH1-positive oligodendroglioma and astrocytoma. NeuN was focally positive in the OLC in 35% of DNT and 37% of dGNT. Synaptophysin was strongly positive in the OLC in 12% of DNT and focally positive in 25%; it was not positive in dGNT in this series. MAP2 was strongly positive in OLC in 50% and focally positive in OLC in 25% of DNT and focally positive in 75% of dGNT. The gangliogliomatous regions showed strong labelling for synaptophysin but variable positivity with NeuN. These findings are in keeping with previous reports. ER1, citrate-based antigen retrieval reagent 1 (Leica Biosystems, UK); ER2, EDTA-based antigen retrieval reagent 2 (Leica Biosystems, UK).
