# Supplementary material for: Pathology–MRI Correlations in Diffuse Low-Grade Epilepsy Associated Tumors
Source: J Neuropathol Exp Neurol. 2017 Oct 12;76(12):1023–33. doi: 10.1093/jnen/nlx090 (PMC5939705; doi:10.1093/jnen/nlx090)
Supplement: Supplementary Data [file nlx090_supp.zip › nlx090_Supptable2.doc]

**Supplemental Table 2. Features Extracted on Review of the Cases and Used for the Classification of DNT Subtypes and Diffuse Glioneuronal Tumor**

| FEATURES RECORDED ON CASE REVIEW | |
| --- | --- |
| MRI features  Location Size  Shape ; specifically if ‘wedge shaped’ Demarcation/delineation : Well defined or infiltrative  Bubbly appearance  Grey matter involvement Cortical expansion  Cortical atrophy with prominent overlying CSF space  Grey-white matter demarcation sharp Multi gyral involvement  Subcortical white matter involvement  Signal in T2 (hypo, iso or hyperintense) Signal in T1 (hypo, iso or hyperintense) Signal in FLAIR  Cystic degeneration Multicystic appearance Calcification Haemorrhage  Bone remodelling/deformation Mass effect  Perilesional signal abnormality (white matter and cortex)  Contrast enhancement Relationship to ventricle Presence of ‘tail sign’  Presence of distinct area of cortical dysplasia | Histological features  Localisation and involvement of anatomical regions Cystic macroscopically  Cystic microscopically Glioneuronal element  Nodular architecture with normal cortex between nodules  Nodules of cells within tumour infiltrative region Diffuse growth pattern predominating Aggregates of abnormal ganglion cells present Calcification  Haemorrhage or Pigmentation  Eosinophilic granular bodies/Rosenthal fibres Rosette formations  Extent of lesion (number gyri involved)  White matter degeneration (rarefaction - reduction in myelinated axons and gliosis/ cavitation)  Necrosis/ mitosis  Adjacent cortex  Perilesional cortex - layer I hypercellular  Perilesional cortex - small intracortical aggregates or tumour cells (usually CD34 positive)  Dyslamination- radial or columnar, away from tumour infiltration zone  Immunohistochemistry labelling / Molecular pathology CD34 1p/19qLOH  GFAP BRAF fusion  MAP2 IDH1 mutation  NeuN BRAF mutation  Ki67 ATRX mutation |

**Supplemental Table 2**. Features extracted on review of the cases and used for the classification of DNT subtypes and diffuse glioneuronal tumour.

.
